# Cost-effectiveness analysis of the prevention of mother-to-child transmission of HIV

**DOI:** 10.1186/s40249-022-00983-z

**Published:** 2022-06-15

**Authors:** Shui-ling Qu, Ai-ling Wang, Hong-mei Yin, Jin-qi Deng, Xiao-yan Wang, Ye-huan Yang, Xiao-ping Pan, Tong Zhang

**Affiliations:** 1 Chinese Center for Disease Control and Prevention, 155 Changbai Road, Changping District, Beijing, 102206 China; 2 Present Address: National Center for Women and Children’s Health, Chinese Center for Disease Control and Prevention, No. 12 Dahuisi Road, Haidian District, Beijing, 100081 China; 3 Capital Institute of Pediatrics, No. 2 Yabao Road, Chaoyang District, Beijing, 100020 China

**Keywords:** Cost-effectiveness, PMTCT, HIV, Benefit–cost ratio, Individual cost

## Abstract

**Background:**

The number of HIV-positive pregnant women accounted for about 10% of China’s total over the past few years in Liangshan Prefecture, Sichuan province in China. Although cost-effectiveness of the PMTCT of HIV have been evaluated in other previous studies, no specific study has been conducted in Liangshan prefecture, nor has the expenses paid individually by HIV-positive pregnant women been included. The purpose of this study was to evaluate both the short-term and long-term cost-effectiveness of PMTCT of HIV in Liangshan Prefecture from the social perspective.

**Methods:**

From December 2018 to January 2019, individual expenses and the other costs were collected: individual expenses of 133 recruited HIV-positive pregnant women registered in the National Information System of Prevention of Mother-to-Child Transmission of HIV, Syphilis, and HBV, and the other costs from local maternal and child healthcare hospitals, Centers for Disease Control and Prevention, and general hospitals. The costs, the number of pediatric infections averted from being HIV infected were analyzed. And, Life years gained by pediatric infections averted were calculated by using a life table. Besides, Direct benefit was calculated through a Markov mode. Furthermore, One-way sensitivity analysis was conducted for key variables affecting the benefit–cost ratio.

**Results:**

The estimated number of pediatric infections averted was 164.The total cost was USD 114.1 million, including direct medical costs, direct non-medical costs, and indirect costs, which were USD 54.2 million, USD 53.4 million, and USD 6.5 million, respectively. 630.6 person-years discounted to 2017 were gained at a 3% annual rate, and cost per life year gained was USD 1809.50. Direct benefits were USD 198.4 million, indirect benefits USD 82.5 million, and the benefit–cost ratio was 1.5. The sensitivity analysis showed that if PMTCT costs hypothetically ranged from USD 85.6 million to USD 142.6 million, benefit–cost ratio would vary from 1.0 to 2.3.

**Conclusions:**

PMTCT of HIV in Liangshan Prefecture was very cost-effective. It was a great economic burden of PMTCT on HIV-positive pregnant women and their families to take individual expenses. Therefore, it could be suggested that individual expenses should be covered as much as possible by different types of financing.

## Background

In 2010, World Health Organization (WHO) recommended maternal Zidovudine(AZT) + infant antiretroviral(ARV) prophylaxis (Option A) and maternal triple ARV prophylaxis(Option B) for treating pregnant women and preventing HIV infection in infants [1], and the third option (Option B+) was recommended by WHO in 2012 [2], in which all pregnant women living with HIV should be offered lifelong triple antiretroviral therapy (ART), regardless of their CD4 + count. Conventionally, every PMTCT scheme includes HIV laboratory testing for all pregnant women and children, maternal ART, and pediatric ART.

The risk of mother-to-child transmission (MTCT) of HIV ranges from 14% to 48% in the absence of any intervention [3], and it could be reduced to less than 2% with effective interventions during the periods of pregnancy, delivery, and breastfeeding [4]. In China, the MTCT rate decreased from 34.8% under no government intervention (before 2005) to 3.6% in 2020 [5], while the number of HIV-positive pregnant women in Liangshan Prefecture of Sichuan province accounted for about 10% of China’s total in the past few years, and the MTCT rate was 3.44% which remained higher than the national average in 2020 [5]. There were several factors influencing the Prevention of mother-to-child transmission (PMTCT) of HIV in Liangshan Prefecture, such as traffic inconvenience, family economic disadvantage, and poor knowledge of hazard of MTCT of HIV [6]. In view of this, a lot of financial and human resources were invested sufficiently by the Chinese government for PMTCT of HIV including ART, early infant diagnosis (EID) of HIV, HIV testing reagents consisted of preliminary screening testing and reinspection testing, laboratory consumables, and laboratory equipment, etc. In Liangshan Prefecture, interventions for PMTCT of HIV were implemented by several related local institutions, mainly including maternal and child healthcare hospitals (MCH), the Center for Disease Control and Prevention (CDC), and general hospitals [7].

It was showed that the PMTCT of HIV was cost-effective in previous studies [8–10]. And Peslie Gibson Ngambi et al. [11] reviewed seven model-based studies on the cost-effectiveness of lifelong ART. The studies above were all model-based studies that the cost and effectiveness were simulated instead of survey. In one of the studies in China, costs were estimated with budget on the health economic evaluations of PMTCT of HIV in Dehong prefecture [12], and deterministic models for simulating a cohort was developed for examining cost-effectiveness of option B+ in PMTCT of HIV in Yunnan Province [13]. However, the model-based studies ignore discrepancies or differences in different settings. Despite the actual situation in reality that these kinds of surveys could discover, they cannot demonstrate the outcomes in the long-term. And there still have been no studies that had individual expenses included in the cost-effectiveness of PMTCT of HIV, to our knowledge. What’ more, no research on the cost-effectiveness of PMTCT of HIV was found online in Liangshan Prefecture. Therefore, according to WHO guide to cost-effectiveness analysis [14], combined with survey and model-based study, this study was designed to evaluate both the short-term and long-term cost-effectiveness of PMTCT of HIV in Liangshan Prefecture from the perspective of society.

## Methods

### Study design

For the perspective of the study was society, all PMTCT costs were incorporated no matter who paid for them. Based on the results of pilot survey, funding channels of PMTCT of HIV included Chinese central government, Sichuan provincial government, Liangshan Prefecture government, counties governments, MCHs, non-governmental organization (NGO), and individuals as well. The expenditure occurred in MCHs, CDCs, general hospitals, and by individual. All costs were collected from institutions above and individuals through two different kinds of questionnaires.

To collect the costs of PMTCT of HIV and the effectiveness data in 2017 was the study conducted from December 2018 to January 2019. Based on high-, medium-, and low-level HIV prevalence rates, the annually reported prevalence rates of HIV-positive pregnant women in the 17 counties of Liangshan prefecture was 0.5–3.2%, 0.1–0.4%, and 0.0–0.1%, respectively. For each prevalence level, two counties were sampled based on purpose sampling for collecting cost data from MCHs, CDCs, and general hospitals.

To collect individual cost, HIV-positive pregnant women registered in the National Information System of PMTCT of HIV, Syphilis, and HBV, was recruited to local clinic for questionnaire survey one by one. Individual costs included meals due to hospital visits, transportation due to hospital visits, infant formula, hospitalization and medical cost during pregnancy and in labor. Loss of workday income due to hospital visits were not included, considering the low level of daily income of Liangshan residents. The total individual costs in Liangshan were calculated by multiplying the average individual cost from survey by the sampling weight.

To estimate the sample size, the individual indirect-costs were set as standard deviation (σ) at USD 74. 1, the allowable error (δ) was set as USD 14.8, and ${\mu }_{\alpha }$($\alpha =0.05$) was 1.96. The parameter above for sample size calculation were estimated based on the local consumption level. The formula for calculating sample size was as follows:

$$n={\left(\frac{{u}_{\alpha }\sigma }{\delta }\right)}^{2},$$

the estimated minimum sample size of HIV-positive pregnant women (*n*) was obtained as 96.

And another method for estimating the sample size was cited, the criterion of sample size for pharmacoeconomic evaluation. The criterion suggested that the minimum sample size of each group should not be less than 100 cases for a study on high-quality pharmacoeconomic evaluation [15].

### Costing

The costs were divided into three parts according to their traceability: direct medical costs, direct non-medical costs, and indirect costs. Direct medical costs covered the cost of drugs for ART and prophylactic, EID of HIV, HIV preliminary screening testing reagents, HIV reinspection testing reagents, laboratory consumables, hospitalization, and laboratory equipment. Direct non-medical costs covered the salary of those who offered intervention services; individual transportation, individual meals, infant formula; and financial aid for pregnant women. Indirect costs included staff training expenses, propaganda expenses, office expenses, and office equipment expenses.

According to Chinese Government Accounting Standard No. 3—Fixed Assets, the useful lifespan was set as 15 years for office furniture, 10 years for lab equipment, 6 years for office equipment, and 5 years each for micropipettor. The scrap value rate was set as 0% for the micropipettor and 5% for the other fixed assets [16]. The annual depreciation of fixed assets was determined using the straight-line method:

$$Annual \, Depreciation = Cost \, of \, Asset* \, \left( {{1 }{-}Scrap \, Value \, Rate} \right)/Useful \, Life.$$

### Cost-effectiveness analysis

#### Effectiveness analysis

In this study, the number of pregnant women tested for HIV (*N*_*1*_) and the number of HIV-positive pregnant women (*N*_2_) were analyzed. To calculate the number of pediatric infections avoided HIV infection from their mothers (*N*_*3*_), *N*_2_ was multiplied by the difference between the rate (34.8%) without any intervention in China and the current rate (9.0%). The formula for calculating *N*_*3*_ was as follows:

$$N_{3} = N_{2} *(34.8 - 9.0\% ).\,$$

#### Health utility analysis

The life years (LYs) gained by *N*_*3*_ were calculated as a measure of health utility using a life table. Here, 76.9 years of age—the life expectancy of the people in Sichuan province in 2017, and age-specific mortality rate from the sixth national census of China were referenced [17]. The cost utility ratio (CUR) was used to measure the cost per LY gained, as follows:

$$CUR= \frac{C}{U},$$

where *C* was the cost of PMTCT, and *U* was the number of LYs gained by *N*_*3*_*.*

#### Cost-benefit analysis

**Fig. 1:**
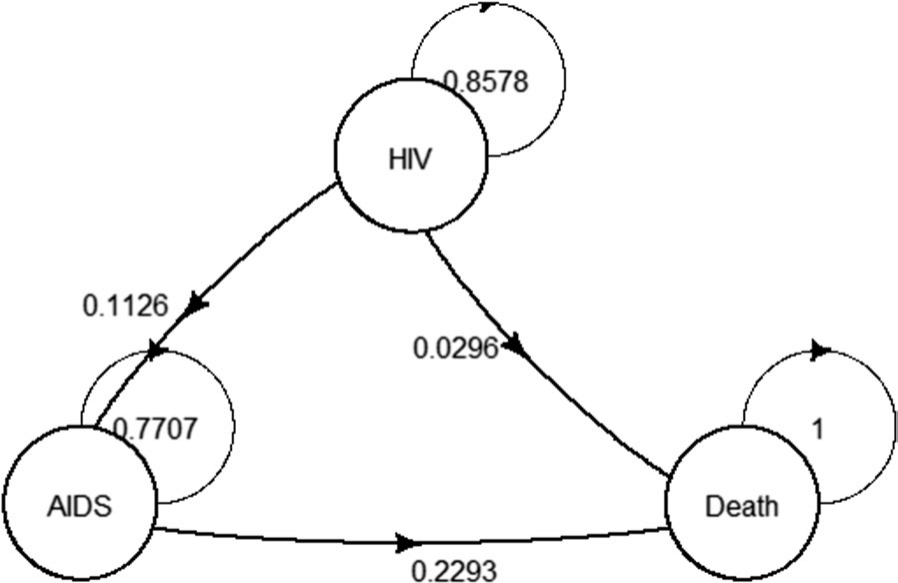
Markov model of HIV/AIDS for ART. *AIDS* acquired immunodeficiency syndrome, *ART* antiretroviral therapy, *HIV* human immunodeficiency virus

The benefits from PMTCT were of two types: direct benefits and indirect benefits. Direct benefit was defined as saving the cost of ART for *N*_*3*_, and it was calculated based on a Markov model with the statistical software R-4.1.1(W. N. Venables, D. M. Smith and the R Core Team) package heemod. For creating the Markov model, it was assumed that the disease process of HIV/AIDS had the following health states: HIV state, AIDS state, and the absorbing state of death. It was also assumed that life expectancy of HIV-infected pediatric from mother was 25 years according to the literature [18], and that the cycle length of the Markov model was one year. The costs after 2017 were discounted to 2017 at the rate of 3% annually (Fig. 1).

The indirect benefit, defined as economic value created by pediatric infections averted, was calculated by multiplying the estimated LYs by age-weighted productivity GDP per capita based on the human capital theory. Age-weighted productivity of those aged 0–14, 15–44, 45–59, and above 60 years is 0.15, 0.75, 0.8, and 0.1, respectively [27].

The benefit–cost ratio (BCR), defined as the proportion of net output to input economically, was considered as the total net benefit per cost of PMTCT. It was calculated as follows:

$$BCR=\frac{\sum b}{\sum c}=\frac{\sum \left(Direct benefit+Nodirect benefit\right)-Cost of PMTCT}{Cost of PMTCT}.$$

### Sensitivity analysis for BCR

One-way sensitivity analysis was conducted for key variables to which BCR were expected to be sensitive. It was assumed that costs of PMTCT could vary by ± 25%. The range of transition probabilities and costs of ART in the Markov model were between lower and upper in Table 1, while the life expectancy of pediatric infections averted ranged from 15 to 35 years, and the discount rate ranged between 0 and 10%. The life expectancy in Liangshan ranged from 60 to 85 years, and its GDP per capita ranged from USD 1295.8 to USD 8393.9 (Table 2).

**Table 1:** Markov model parameters of HIV/AIDS for ART

Parameter	Value
Initial distribution of HIV/AIDS (%)	
HIV state	100.0%
AIDS state	0.0%
Transition probabilities per year (%)	
HIV state > AIDS state [mean (min–max)]	11.3 (9.1–13.2)^a^ [19, 20]
HIV state > Death [mean (min–max)]	3.0 (0.5–3.7)^a^ [19–22]
AIDS state > Death [mean]	22.9^b^ [23]
Costs of ART (USD/person-year)	
HIV state [mean (min–max)]	967.9 (533.0–1254.7) [24]
AIDS state [mean (min–max)]	3036.1 (2749.2–3323.0) [25, 26]
Discount [mean (min—max)]	3% (0–10%)

*HIV/AIDS* human immunodeficiency virus/acquired immune deficiency syndrome, *ART* antiretroviral therapy

^a^Transformed by the mortality rate per 100 people

^b^Average value over years

**Table 2:** Cost-effectiveness of PMTCT of HIV in Liangshan Prefecture, Sichuan province in China

Items	Value
Costs (USD in million)	
PMTCT (HIV test, ART, etc.)	114.1
Effectiveness	
No. of pregnant women preliminary tested for HIV	34,991
No. of identified HIV + women	663
No. of HIV + women volunteering to terminate pregnancy	28
No. pediatric infections averted	164
Life years gained (person-years)	630.6
Direct benefits (USD in million)	198.4
Indirect benefits (USD in million)	82.5
Cost-effectiveness	
Cost per pregnant women preliminary screening tested for HIV (USD)	3.5
Cost per pregnant women reinspected for HIV (USD)	296.6
Cost per pediatric infections averted (USD)	6957.9
Cost utility ratio	1809.5
Benefit–cost ratio	1.5

*PMTCT* prevention of mother-to-child transmission, *HIV* human immunodeficiency virus, *ART* antiretroviral therapy

## Results

### Basic information

In 2017, 34,991 pregnant women registered in the National Information System of Prevention of Mother-to-Child Transmission of HIV, Syphilis, and HBV, were tested for HIV, and 663 of them were HIV-positive after reinspection testing. In this study, 133 HIV-positive pregnant women among them were recruited to fill out the individual questionnaire. Besides, six MCHs, six CDCs, and six General Hospitals were investigated.

### Costs

Costs collected from institutions plus the individual costs were the total cost of PMTCT in 2017 at USD 114.1 million. The costs were from fiscal expenditure, individual expenses, MCHs, and NGOs, which were USD 77.8 million, USD 20.8 million, USD 13.7 million, and USD 1.9 million, accounting for 68.2%, 18.3%, 12.0%, and 1.6%, respectively.

**Fig. 2:**
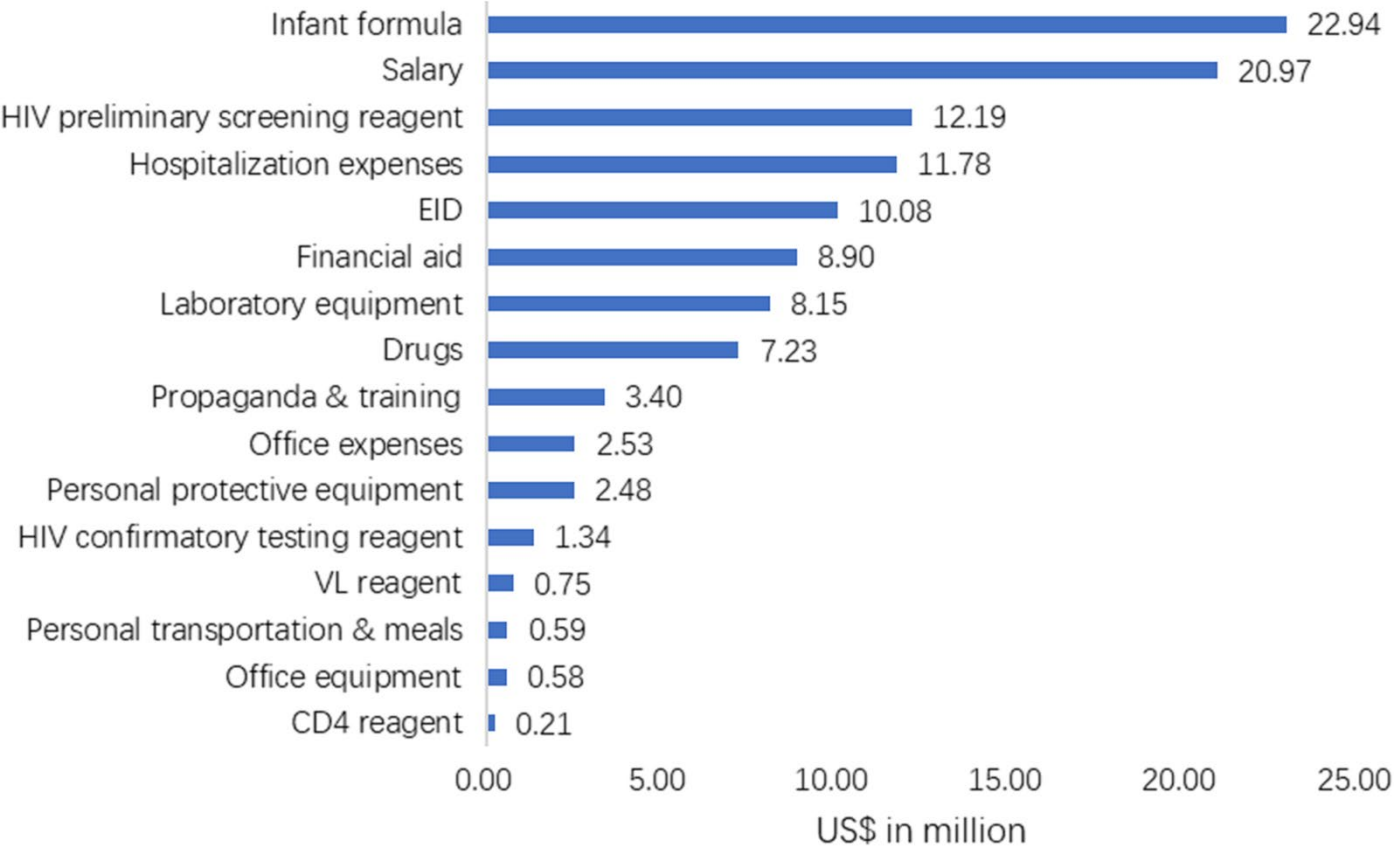
The costs structure of PMTCT of HIV in Liangshan Prefecture, Sichuan province in China. *CD4* CD4 lymphocyte count, *EID* early infant diagnosis, *HIV* human immunodeficiency virus, *VL* viral load

The direct medical costs, direct non-medical costs, and indirect costs, were USD 54.2 million, USD 53.4 million, and USD 6.5 million, accounting for 47.5%, 46.8%, and 5.7%, respectively. For more details, see Fig. 2.

The median costs per HIV-positive pregnant women were USD 319.9, of which median meal costs, transportation costs, costs of infant formula, and hospitalization expenses during pregnancy and in labor per HIV-positive pregnant women were USD 4.4, USD 4.4, USD 133.3, and USD 177.7, respectively.

### Cost-effectiveness

In 2017, the estimated number of pediatric infections with HIV from their mothers would have been 221 free from any intervention. Under present interventions, the estimated number decreased by 57, with 164 averted. To avert one infant infection, USD 6957.9 was needed on average. LYs gained from pediatric infections averted were 630.6 person-years, and the cost per LY gained from PMTCT was USD 1809.5, discounted to 2017 at the rate of 3% annually. The direct and indirect benefits were USD 198.4 million and USD 82.5 million, respectively. The BCR, total net benefit per cost of PMTCT, was 1.5.

### Sensitivity analyses for BCR

**Fig. 3:**
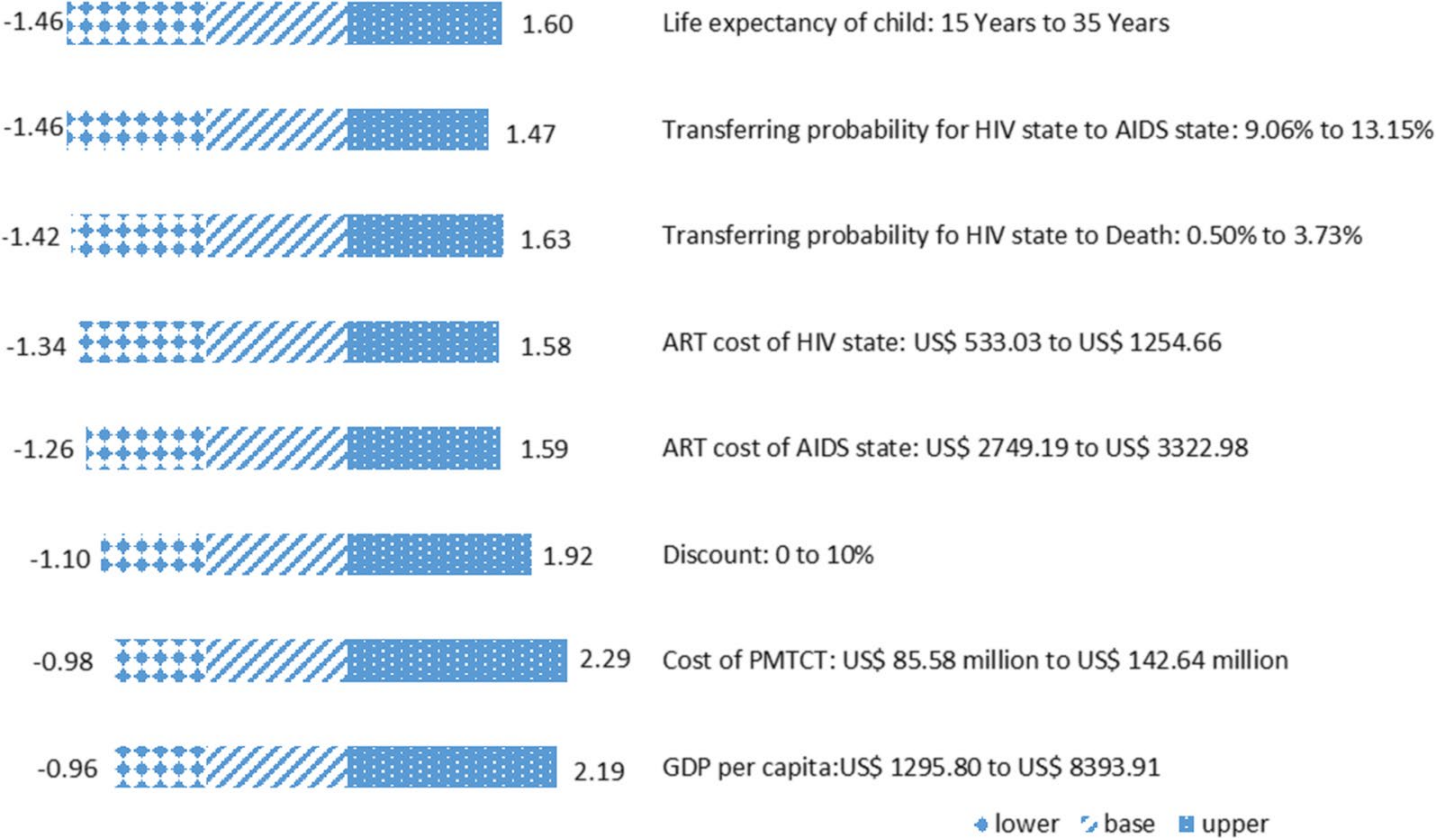
The effects of changes in variables on the benefit–cost ratio. *AIDS* acquired immunodeficiency syndrome, *ART* antiretroviral therapy, *GDP* gross domestic product, *HIV* human immunodeficiency virus, *PMTCT* prevention of mother-to-child transmission

Results of the one-way sensitivity analysis showed that the costs of PMTCT had the most effect on BCR. If, hypothetically, the costs of PMTCT ranged from USD 85.6 million to USD 142.6 million, the BCR would vary from 1.0 to 2.3. Moreover, hypothetically, a varying GDP per capita could have a significant impact on BCR, with BCR varying from 1.0 to 21.9. The results also showed that life expectancy of pediatric infections averted had nearly no effect on BCR. For more details, see Fig. 3.

## Discussion

The Statistics Review of Liangshan showed that this prefecture’s GDP per capita in 2017 was USD 4542.3 [28], which was less than that for Sichuan province’s USD 6613.2 and Beijing’s USD 19105.1 [29]. The per capita disposable income and per capita living expenditure of rural residents in Liangshan Prefecture were USD 1690.7 and USD 1293.6, respectively in 2017 [28]. On condition that the per capita disposable income was subtracted by per capita living expenditure, only USD 450.4 was left. This study found that the average individual cost for HIV-positive pregnant women was USD 319.9 which was little less than USD 450.4. It was showed in one of previous studies that HIV-positive pregnant women would lose motivation to seek PMTCT services if the individual expenses exceeded what she could afford [6]. Accordingly, from this study, it could be recommended that individual expenses should be covered as much as possible by different types of financing. The financing channels for hospitalization expenses could be medical insurance, donate from NGOs and individual, minimum living standard security system. Infant formula manufacturers also may be a good financing channels for infant formula. If so, an additional USD 148,108.7 per year should be expected and invested to cover all of the personal expenses in Liangshan Prefecture, which would further reduce the MTCT rate.

To our knowledge, it is the first study that conducts a cost-effectiveness analysis of PMTCT of HIV in Liangshan Prefecture, where the annual number of HIV-positive pregnant women have accounted for about 10% of the total in China for the past few years. Also, it is the first study to incorporate individual expenses for PMTCT into a cost-effectiveness analysis of PMTCT of HIV in China [12, 13, 30]. This study is critical for an impoverished mountainous area like Liangshan Prefecture.

Under the interventions of PMTCT of HIV, 663 pregnant women were tested HIV-positive in 2017 in Liangshan Prefecture. The average cost for these pregnant women and their children was USD 172,109.9. A previous study in Ethiopia showed that the average cost per pregnant woman-infant pair per year ranged from USD 319 to USD 1099 in urban health facilities with high prevalence of HIV infection; however, in rural health facilities with low prevalence of HIV infection, the costs ranged from USD 220 to USD 383 [31]. The reason why cost per pregnant woman-infant pair in Liangshan was higher than that in Ethiopia could be that HIV prevalence in Ethiopia was much higher than in Liangshan. Another study in Uganda showed that the average unit cost of Option B + services per mother-infant pair was USD 441.9 [32], which did not cover infant formula, VL reagent, propaganda expenses, and financial aid, etc. And these differences suggested that it was important to consider which items should be covered by cost of PMCTC in order to optimize the allocation of resources. Cost per pediatric infections averted in the study was USD 6957.9, which was higher than that of USD 3,388.9 in Henan [33], USD 409.2 in Xinjiang [34], and lower than that of USD 18,005 in Yunnan [35], the three provinces in China. In the study, the CUR was USD 1809.5 per LY gained, which was slightly more than that of USD 1160 in Cape Town, South Africa [36]. It must be stated that the health utility index was life year in this study while it was QALY in the latter study. The estimates in a previous study showed that each LY was valued at around three times the annual earnings [37]. The CUR in this study was less than USD 4542.3 [28], the GDP per capita in Liangshan. According to the WHO’s criterion that each healthy life gained at a cost less than the GDP per capita is defined as very cost-effective [14], the PMTCT of HIV in Liangshan Prefecture was also very cost-effective.

This study investigated institutional and individual costs and also highlighted the costs, CUR, and BCR, but the study had several limitations. The parameters of the Markov model were cited from the public data, not survey data, and so the publication bias was unavoidable. Besides, though a complete random sampling method would be more appropriate for this study, these women were recruited based on the occasional sampling method due to the particularity of HIV positive pregnant women and the attempts to protect their privacy. Still, the individual expenses might have some bias. Another shortcoming is that loss of workday income due to hospital visits were not included, given the low daily income of Liangshan residents. If so, the individual costs would increase.

## Conclusions

The PMTCT of HIV in Liangshan Prefecture was very cost-effective. It was a great financial burden of PMTCT on HIV-positive pregnant women and their families to take individual expenses. And, it could be suggested that individual expenses should be covered as much as possible by different types of financing.

## Data Availability

The dataset supporting the conclusion of this article is available upon reasonable request from the corresponding author.

## Abbreviations

AIDS: Acquired immunodeficiency syndrome
ART: Antiretroviral therapy
ARV: Antiretroviral
BCR: Benefit–cost ratio
CDC: Center for Disease Control and Prevention
CUR: Cost utility ratio
EID: Early infant diagnosis
GDP: Gross domestic product
HIV: Human immunodeficiency virus
LY: Life year
MCH: Child health care hospital
MTCT: Mother-to-child transmission
PMTCT: Prevention of mother-to-child transmission
VL: Viral load
WHO: World Health Organization

## References

[1] WHO. Antiretroviral drugs for treating pregnant women and preventing HIV infection in infants. Recommendations for a public health approach (2010 version). https://www.who.int/publications/i/item/9789241599818. Accessed 1 May 2022. 26180894

[2] WHO. WHO HIV update: global epidemic, progress in scale up and policy uptake. https://www.who.int/hiv/data/en/. Accessed 24 Feb 2019.

[3] John GC, Kreiss J (1996). Mother-to-child transmission of human immunodeficiency virus type 1. Epidemiol Rev.

[4] Ishikawa N, Newman L, Taylor M, Essajee S, Pendse R, Ghidinelli M (2016). Elimination of mother-to-child transmission of HIV and syphilis in Cuba and Thailand. Bull World Health Organ.

[5] Wang A, Song L (2021). Protecting the beginning of Life and Safeguarding Health of Maternal and child—review and prospect of the Prevention of mother-to-child transmission of HIV, syphilis and Hepatitis B in China in the past 20 years. Chin J AIDS STD.

[6] Yang A, Zhou Y, E M, Song X, Nie S, Zhang T, et al. Qualitative study on affecting factors of prevention of mother-to-child HIV transmission in Liangshan Yi Area. Chin Prim Health Care. 2013;27(08):63–65. (In Chinese)

[7] Wang AL, Qiao YP, Wang LH, Fang LW, Wang F, Jin X (2015). Integrated prevention of mother-to-child transmission for human immunodeficiency virus, syphilis and hepatitis B virus in China. Bull World Health Organ.

[8] Kuznik A, Lamorde M, Hermans S, Castelnuovo B, Auerbach B, Semeere A (2012). Evaluating the cost-effectiveness of combination antiretroviral therapy for the prevention of mother-to-child transmission of HIV in Uganda. Bull World Health Organ.

[9] Ishikawa N, Dalal S, Johnson C, Hogan DR, Shimbo T, Shaffer N (2016). Should HIV testing for all pregnant women continue? Cost-effectiveness of universal antenatal testing compared to focused approaches across high to very low HIV prevalence settings. J Int AIDS Soc.

[10] Fasawe O, Avila C, Shaffer N, Schouten E, Chimbwandira F, Hoos D (2013). Cost-effectiveness analysis of option B+ for HIV prevention and treatment of mothers and children in Malawi. PLoS ONE.

[11] Ngambi PG, Kalungia AC, Law MR, Kalemeera F, Truter I, Godman B, Munkombwe D (2017). Evidence on the cost-effectiveness of lifelong antiretroviral therapy for prevention of mother-to-child transmission of HIV: implications for resource-limited countries in sub-Saharan Africa. Exp Rev Pharmacoecon Outcomes Res.

[12] Shan D, Wang J, Duan S, Guo Y, Tang S, Yang Y (2015). A study on the health economic evaluations of prevention of mother-to-child HIV transmission in Dehong prefecture, Yunnan province, China from 2004 to 2013. Chin J Prevent Med.

[13] Wang X, Guo G, Zheng J, Lu L (2019). Cost-effectiveness of option B+ in prevention of mother-to-child transmission of HIV in Yunnan Province, China. BMC Infect Dis.

[14] WHO. WHO Guide to cost-effectiveness analysis. https://apps.who.int/iris/bitstream/handle/10665/42699/9241546018.pdf?sequence=1. Accessed 1 May 2022.

[15] Guoen L (2015). China guidelines for pharmacoeconomic evaluations and manual.

[16] Zhang H (2012). Accounting and tax differences of salvage value of fixed assets. Juanzong.

[17] Census Office of the State Council of China. Tabulation on 2010 population census of the People’s Republic of China. http://www.stats.gov.cn/tjsj/pcsj/rkpc/6rp/indexch.htm. Accessed 7 Apr 2019 (In Chinese).

[18] Ciaranello AL, Doherty K, Penazzato M, Lindsey JC, Harrison L, Kelly K (2015). Cost-effectiveness of first-line antiretroviral therapy for HIV-infected African children less than 3 years of age. AIDS.

[19] Liao L, Xing H, Su B, Wang Z, Ruan Y, Wang X (2013). Impact of HIV drug resistance on virologic and immunologic failure and mortality in a cohort of patients on antiretroviral therapy in China. AIDS.

[20] Chen J, Zhang M, Shang M, Yang W, Wang Z, Shang H (2018). Research on the treatment effects and drug resistances of long-term second-line antiretroviral therapy among HIV-infected patients from Henan Province in China. BMC Infect Dis.

[21] Yang W. Analysis on survival effect and effect factors after initiating antiretroviral treatment among AIDS patients. Zhengzhou University; 2015. (In Chinese)

[22] Sigaloff KC, Hamers RL, Wallis CL, Kityo C, Siwale M, Ive P (2012). Second-line antiretroviral treatment successfully resuppresses drug-resistant HIV-1 after first-line failure: prospective cohort in Sub-Saharan Africa. J Infect Dis.

[23] Poorolajal J, Hooshmand E, Mahjub H, Esmailnasab N, Jenabi E (2016). Survival rate of AIDS disease and mortality in HIV-infected patients: a meta-analysis. Public Health.

[24] Ju L-H, Zeng G, XU P, Xin Q-Q, Zeng J, LV F. Unit cost of antiretroviral treatment of HIV/AIDS cases. Chin J Public Health. 2013;29(11):1580–1583. (In Chinese)

[25] Guo Z. The direct medical costs analysis of national free Antiretroviral treatment for HIV/AIDS patients. Peking Union Medical College; 2008 (In Chinese).

[26] Li Y, Wu J, Zhang H (2017). Analysis of hospitalization expenses of opportunity infection treatment in AIDS patients. Chin Med Record.

[27] Xiaoming C (2016). Health economics.

[28] Liangshan Bureau of Statistics. 2017 National Economic and Social Development Statistical Bulletin of Liangshan Prefecture. http://tjj.lsz.gov.cn/sjfb/lstjgb/201808/t20180816_1176634.html. Accessed 01 May 2022. (In Chinese)

[29] National Bureau of Statistics of China (2018). China Statistical Yearbook 2018.

[30] Liu S. Health economic evaluation on prevention and control program of mother-to-child HIV transmission in Shenzhen. Huazhong University of Science & Technology, 2011. (In Chinese)

[31] Zegeye EA, Mbonigaba J, Kaye S, Johns B (2019). Assessing the cost of providing a prevention of mother-to-child transmission of HIV/AIDS service in Ethiopia: urban–rural health facilities setting. BMC Health Serv Res.

[32] Mukose AD, Kebede S, Muhumuza C, Makumbi F, Komakech H, Bayiga E (2020). Costs and cost drivers of providing option B+ services to mother-baby pairs for PMTCT of HIV in Health Centre IV Facilities in Jinja District, Uganda. Biomed Res Int.

[33] Xuezhou L (2008). Health economics analysis of implementation of interruption vertical transmission in patients with acquired immunodeficiency syndrome (AIDS). Med Soc.

[34] Yi-ji Y, Tao J, Jin-bao L, Hui-ling X, Si-min B, Xiao-yuan H. Cost-effectiveness analysis of interruption on the mother-to-child transmission in Xinjiang. Chin Health Econ. 2012;31(10):47–8. (In Chinese)

[35] Wang X, Guo G, Zheng J, Lu L (2019). Programmes for the prevention of mother-to-child HIV infection transmission have made progress in Yunnan Province, China, from 2006 to 2015: a cost effective and cost-benefit evaluation. BMC Infect Dis.

[36] Zulliger R, Black S, Holtgrave DR, Ciaranello AL, Bekker LG, Myer L (2014). Cost-effectiveness of a package of interventions for expedited antiretroviral therapy initiation during pregnancy in Cape Town, South Africa. AIDS Behav.

[37] Health WCOM. Macroeconomics and health: Investing in health for economic development. Report of the Commission on Macroeconomics and Health. https://apps.who.int/iris/bitstream/handle/10665/42435/924154550X.pdf;sequence=1. Accessed 1 May 2022.

